# Spinal alignment shift between supine and prone CT imaging occurs frequently and regardless of the anatomic region, risk factors, or pathology

**DOI:** 10.1007/s10143-021-01618-x

**Published:** 2021-08-11

**Authors:** Lars Wessels, Bettina Komm, Georg Bohner, Peter Vajkoczy, Nils Hecht

**Affiliations:** 1 Department of Neurosurgery, Charité-Universitätsmedizin Berlin, Charitéplatz 1, 10117 Berlin, Germany; 2 Department of Neuroradiology, Charité-Universitätsmedizin Berlin, Berlin, Germany

**Keywords:** Computer-assisted spine surgery, Intraoperative imaging, Spinal alignment shift

## Abstract

Computer-assisted spine surgery based on preoperative CT imaging may be hampered by sagittal alignment shifts due to an intraoperative switch from supine to prone. In the present study, we systematically analyzed the occurrence and pattern of sagittal spinal alignment shift between corresponding preoperative (supine) and intraoperative (prone) CT imaging in patients that underwent navigated posterior instrumentation between 2014 and 2017. Sagittal alignment across the levels of instrumentation was determined according to the C2 fracture gap (*C2-F*) and C2 translation (*C2-T*) in odontoid type 2 fractures, next to the modified Cobb angle (*CA*), plumbline (*PL*), and translation (*T*) in subaxial pathologies. One-hundred and twenty-one patients (C1/C2: *n* = 17; C3-S1: *n* = 104) with degenerative (39/121; 32%), oncologic (35/121; 29%), traumatic (34/121; 28%), or infectious (13/121; 11%) pathologies were identified. In the subaxial spine, significant shift occurred in 104/104 (100%) cases (*CA*: *p = .044; *T*: *p = .021) compared to only 10/17 (59%) cases that exhibited shift at the C1/C2 level (*C2-F*: **p = .002; *C2-T*: *p < .016). The degree of shift was not affected by the anatomic region or pathology but significantly greater in cases with an instrumentation length > 5 segments (“∆*PL* > 5 segments”: 4.5 ± 1.8 mm; “∆*PL* ≤ 5 segments”: 2 ± 0.6 mm; *p = .013) or in revision surgery with pre-existing instrumentation (“∆*PL *presence”: 5 ± 2.6 mm; “∆*PL *absence”: 2.4 ± 0.7 mm; **p = .007). Interestingly, typical morphological instability risk factors did not influence the degree of shift. In conclusion, intraoperative spinal alignment shift due to a change in patient position should be considered as a cause for inaccuracy during computer-assisted spine surgery and when correcting spinal alignment according to parameters that were planned in other patient positions.

## Introduction

Computer-assisted spine surgery is rapidly gaining acceptance due to improved accuracy [13, 27, 36], reduced radiation exposure [32, 34, 42], and the promise for better outcomes [6, 43]. An important factor that currently limits widespread implementation is high acquisition and maintenance costs of state-of-the-art intraoperative CT (iCT) or cone-beam CT (CBCT) imaging [6]. To some extent, these costs may be offset by performing image-guided surgery based on preoperative CT imaging alone, where navigated or robotic-assisted screw insertion is achieved by surface matching registration [4, 20, 29, 33, 39]. However, pedicle screw accuracy appears to be lower when image-guidance is based on preoperative instead of intraoperative imaging [24]. This represents a dilemma, because preoperative CT-based navigation and robotic-assisted surgery are meanwhile routinely implemented and remain regularly used, including augmented reality (AR) and machine learning-based applications that continue to rely on preoperative CT data sets [19]. Furthermore, preoperative CT imaging is usually performed with the patient in supine position, whereas posterior pedicle screw instrumentation is performed in prone. This difference in position could result in a sagittal alignment shift with uncertainty for the surgeon and reduced accuracy of computer-assisted surgery based on preoperative CT alone. At closer view, however, a systematic analysis on the occurrence, localization, and risk factor pattern of sagittal spinal alignment shifts due to patient repositioning has not yet been reported. Therefore, the aim of the present study was to analyze the pattern of spinal sagittal alignment shifts between preoperative and intraoperative CT imaging in patients undergoing navigated posterior instrumentation across the entire spine.

## Materials and methods

This retrospective cohort study was approved by the ethics committee of the Charité-Universitätsmedizin Berlin, Germany (EA4/046/16) and included 121 patients (55 female, 66 male) that underwent navigated iCT-based posterior instrumentation in our Department between 2014 and 2017. Informed consent was waived due to the retrospective nature of the study. Consecutive patients were selected according the availability of a preoperative (supine) CT data set in addition to and intraoperative (prone) CT data set that was used for navigated pedicle screw insertion. The influence of patient positioning on the sagittal spinal alignment shift between the preoperative and intraoperative CT was determined with 5 sagittal alignment parameters at the C1/C2 (2 parameters) level and across the remaining subaxial spine (3 parameters). Demographic, clinical, and radiographic data were retrospectively collected and analyzed by an independent observer who was not involved in the patients’ care.

### Image acquisition

For intraoperative image acquisition in prone position, the mobile AIRO iCT scanner (Brainlab AG, München, Germany) was used and the patient was positioned prone. Surgery and intraoperative imaging were performed on a radiolucent, non-hinged, carbon-fiber examination table (TRUMPF Carbon FloatLine, TRUMPF Medizin Systeme GmbH & Co. KG, Saalfeld, Germany). For surgery at the mid-thoracic to cervical level, the patients’ head was fixed in a radiolucent carbon fiber 3-pin head clamp (TRUMPF X-RAY, TRUMPF Medizin Systeme GmbH & Co. KG, Saalfeld, Germany). For spinal navigation, the iCT was connected to an image guidance system with infrared tracking camera (BrainLab Curve™, Brainlab AG, Feldkirchen, Germany) allowing automatic patient/image co-registration and image transfer. After surgical exposure and fixation of the navigation tracking device to a spinous process or the iliac crest, the patient was rotated into the iCT gantry and an iCT navigation scan was performed. In case of revision surgery with presence of a previous instrumentation system, all connecting rods and implants requiring revision were removed prior to the scan. Next, navigated pedicle screw insertion was performed as previously described [17, 18, 38]. The iCT scan was executed by a CT-qualified technical radiological assistant and neuroradiologist and iCT data sets were automatically transferred to our in-hospital Picture Archiving and Communications System (PACS) for analysis.

For preoperative image acquisition in supine position, a Toshiba Aquilion Prime, Toshiba Aquilion One (Canon Medical Systems, Tustin, CA, USA) GE Revolution HD, GE Revolution EVO, GE Revolution CT, and GE LightSpeed VCT (General Electric, Boston, MA, USA) scanners were used.

### Sagittal shift parameters and image analysis

**Fig. 1:**
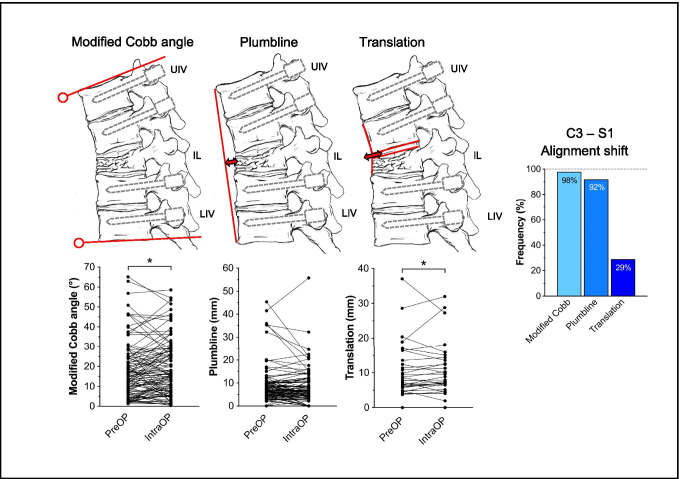
Illustration of the subaxial alignment parameters “modified Cobb angle” (*CA*; left), “plumbline” (*PL*; center), and “translation” (*T*; right). The line graphs illustrate the corresponding sagittal alignment shift between preoperative and intraoperative CT imaging for each patient. The bar graph on the right shows the overall frequency of alignment shift detection for each parameter. UIV, upper instrumented vertebra; LIV, lower instrumented vertebra; IL, index level. *CA*: *p = 0.044; *T*: *p = 0.021; Wilcoxon matched pairs signed rank test

**Fig. 2:**
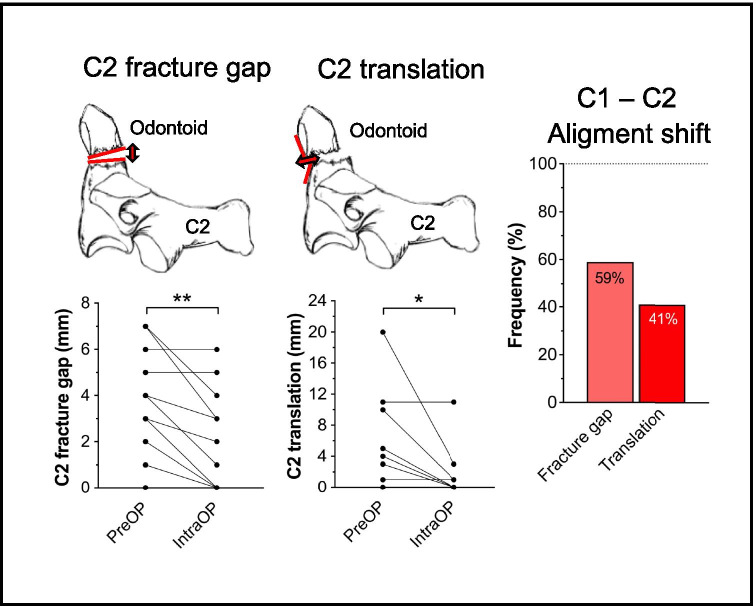
Illustration of the C1/C2 alignment parameters “C2 fracture gap” (*C2-F*; left) and “C2 translation” (*C2-T*; right). The line graphs illustrate the corresponding sagittal alignment shift between preoperative and intraoperative CT imaging for each patient. The bar graph on the right shows the overall frequency of alignment shift detection for each parameter. **p = 0.002 for *C2-F* and *p < 0.016 for *C2-T*; Wilcoxon matched pairs signed rank test

The spinal sagittal shift between preoperative and intraoperative CT data sets was determined with spinal imaging software (Spine Planning – Viewer Ver. 5.1.0.97, Brainlab AG, München, Germany) based on the following parameters: For the subaxial spine, sagittal shift was quantified by measuring a modified Cobb angle (*CA*) between the upper and lower instrumented vertebra, the distance of a plumbline (*PL*) between the upper to the lower instrumented vertebra to the index level of pathology, and the sagittal translation (*T*) at the index level of pathology (Fig. 1). At the C1/C2 level, sagittal shift was quantified by measuring the maximum distance of a C2 (odontoid) fracture gap (*C2-F*) and the sagittal translation of a C2 (odontoid) fracture (*C2-T*) (Fig. 2).

The degree of the alignment shift between corresponding preoperative and intraoperative sagittal CT images at midline was determined in each patient and for each parameter. For subaxial pathologies, alignment shift (*CA*,* PL*, and *T*) was additionally analyzed according to the anatomic region and underlying spinal pathology. In addition, an exploratory analysis of risk factors that might influence the degree of shift was performed.

### Statistical analysis

Descriptive summary statistics are presented as mean ± 95% confidence interval (95%CI), median and interquartile range (IQR), median and range, or percentage, as appropriate. Normal distribution was tested using the Shapiro–Wilk test. For contingency analysis, Fisher’s exact test was used. For comparison of preoperative and intraoperative alignment parameters, a Wilcoxon matched pairs signed rank test was used. For comparison of the degree of shift *(∆CA*,* ∆PL*, and *∆T*) depending on the anatomic region and the underlying pathology, a Kruskal–Wallis test with multiple comparisons and Dunn’s correction was performed. For risk factor analysis, a Mann–Whitney *U*-test was used. All statistics were calculated with GraphPad Prism for Mac (Version 9.0.0, GraphPad Software, San Diego, CA, USA). Statistical significance was set at *p* < 0.05 and all tests were two-sided.

## Results

Between 2014 and 2017, we identified 121 patients that underwent navigated, iCT-based posterior instrumentation with additional availability of a corresponding, preoperative CT scan. Seventeen patients received surgery at the C1/C2 level and 104 patients underwent surgery at the level of the subaxial spine. Baseline demographic data, the instrumented region, the indication for surgery, and the duration of surgery and hospitalization are presented in Table 1.

**Table 1:** Demographics

**Age in years (median, IQR)**	68 (56–76)
**Sex (*n*, %)**	
Male	66 (55%)
Female	55 (45%)
**Weight in kg (median, IQR)**	73.5 (60–90)
**Height in cm (mean ± 95%CI)**	170 ± 1.8
**BMI (median, IQR)**	25 (22–30)
**Instrumented region (*n*, %)**	
C1/C2	17 (14%)
Subaxial cervical	17 (14%)
Cervical-thoracic	18 (15%)
Thoracic	31 (26%)
Thoracic-lumbar	38 (31%)
**Indication for surgery (*n*, %)**	
Degenerative disease	39 (32%)
Tumor	35 (29%)
Trauma	34 (28%)
Infection	13 (11%)
**Surgery duration in minutes (median, IQR)**	231 (189–320)
**Days of hospitalization (median, IQR)**	16 (9.5–27)

In the subaxial spine, sagittal alignment shift between preoperative and intraoperative CT imaging was noted in 104/104 cases (100%) and most frequently detected by *CA* (98%), followed by *PL* (92%) and *T* (29%). Significant shift between preoperative and intraoperative imaging was determined with *CA* and *T* (*CA*: *p = 0.044; *T*: *p = 0.021; Fig. 1). For surgery at C1/C2, alignment shift was only observed in 10/17 cases (59%), and most often detected by *C2-F* (59%), followed by *C2-T* (41%). Significant shift was noted with both parameters (*C2-F:* **p = 0.002; *C2-T:* *p < 0.016; Fig. 2).

**Fig. 3:**
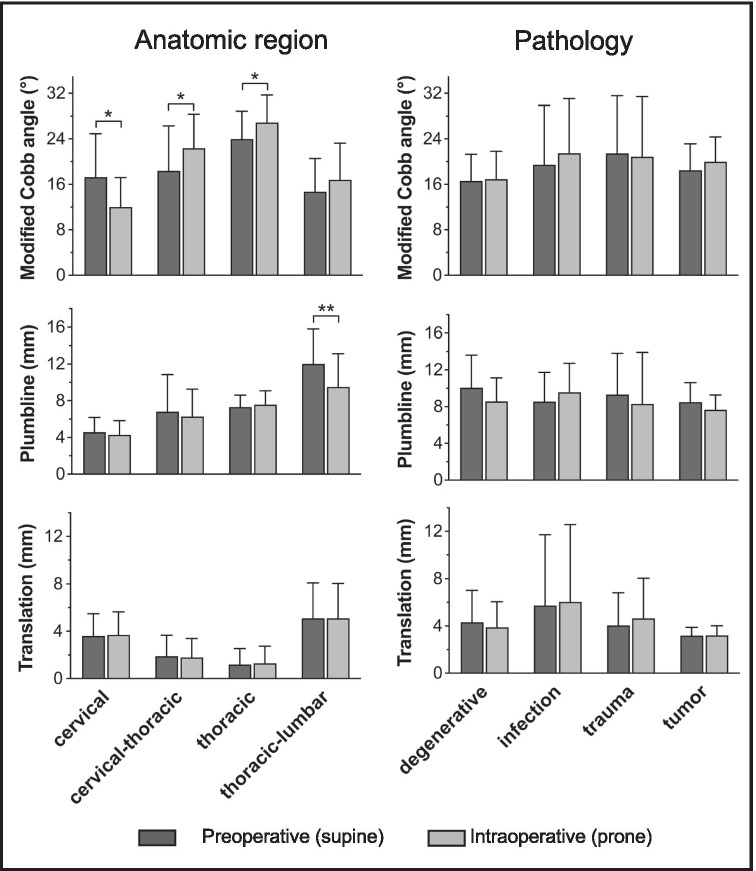
Bar graphs showing the comparison between preoperative and intraoperative subaxial spinal alignment as determined by the modified Cobb angle (*CA*), plumbline (*PL*), and translation (*T*) according to the anatomic region of instrumentation (left panels) and the underlying pathology (right panels). *CA*: *p = 0.049 for cervical, *p = 0.048 for cervical-thoracic, *p = 0.048 for thoracic; *PL*: **p = 0.003 for thoraco-lumbar; Wilcoxon matched pairs signed rank test

For alignment analysis depending on the region of instrumentation, *CA* was best suited for shift detection at the cervical-thoracic region (*CA*: *p = 0.049 for cervical, *p = 0.048 for cervical-thoracic, *p = 0.048 for thoracic) and *PL* at the thoraco-lumbar spine (*PL*: **p = 0.003 for thoraco-lumbar; Fig. 3). Interestingly, none of the parameters detected significant shift when analyzing alignment depending on the pathology (Fig. 3, right panels) and no difference was found in the degree of shift between anatomic regions or the underlying pathology (*p* > 0.05 for individual comparisons of the anatomic region and underlying pathology for *∆CA*,* ∆PL*, and *∆T*).

**Fig. 4:**
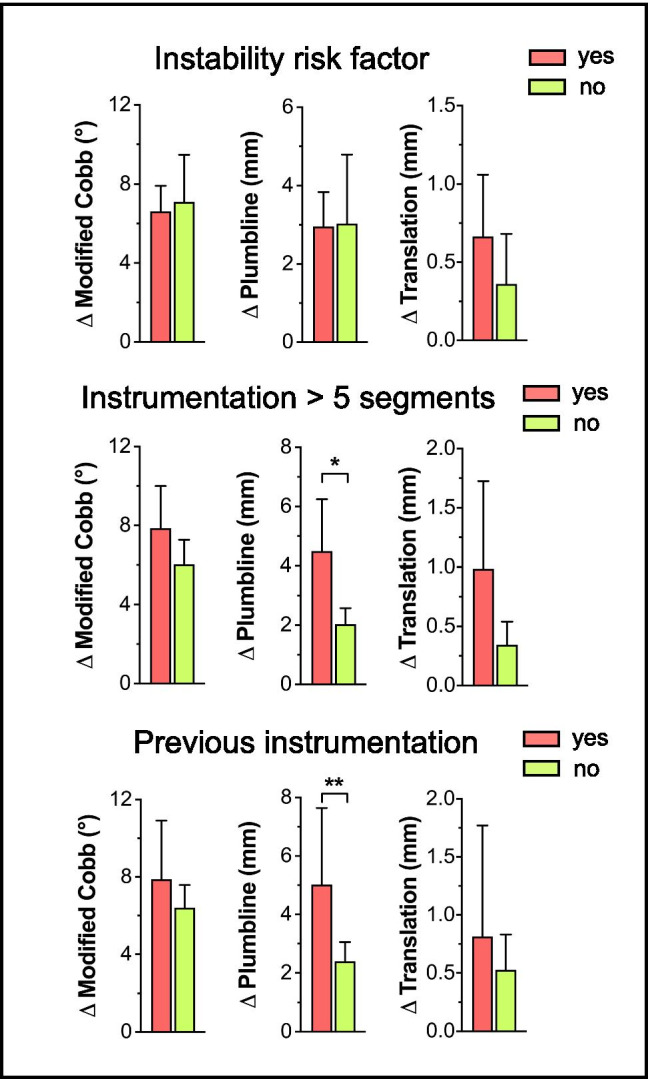
Bar graphs showing the exploratory risk factor analysis according to the presence (red) or absence (green) of (i) a morphological instability risk factor, (ii) an instrumentation length > 5 segments, and (iii) a previous instrumentation. In cases requiring an instrumentation > 5 segments and revision surgery with presence of a previous instrumentation, a significantly greater plumbline shift (∆*PL*) was noted. Previous instrumentation: **p = 0.007; Instrumentation > 5 segments: *p = 0.013; Mann–Whitney *U*-test

Next, we performed an exploratory analysis to identify factors that could be associated with a higher degree of shift, as listed in Table 2. As expected, subaxial cases more often had preexisting instrumentation at the pathological index level (*p = 0.040) and received a longer construct than surgeries at C1/C2 (*p < 0.0001; Table 2). However, the presence of a morphological instability risk factor (Table 2), as well as the rate of complications (including secondary screw revision surgery) did not differ between subaxial and C1/C2 cases (Table 3). In order to obtain a representative view, we therefore decided to focus our analysis on subaxial cases only. Here, *PL* identified a significantly greater alignment shift in cases requiring an instrumentation length > 5 segments (“*∆PL* > 5 segments”: 4.5 ± 1.8 mm; “*∆PL* ≤ 5 segments”: 2 ± 0.6 mm; *p = 0.013) or in cases with preexisting instrumentation (“*∆PL *presence”: 5 ± 2.6 mm; “*∆PL *absence”: 2.4 ± 0.7 mm; **p = 0.007). Unexpectedly, the presence of a morphological spinal instability risk factor did not influence the degree of shift, regardless of the applied parameter (Fig. 4).

**Table 2:** Risk factors of instability

	**Total**	*C1/C2*	*Subaxial spine*	
**Instrumentation present at index level (*n*, %)**	23 (19%)	-	23 (19%)	***p* = 0.040**
** ≥ 1 instability risk factor (*n*, %)**	94 (78%)	15 (88%)	79 (76%)	*p* = 0.356
Osteoporosis	19 (16%)	1 (6%)	18 (17%)	
Rheumatoid arthritis	5 (4%)	3 (18%)	2 (2%)	
Bechterew’s disease	6 (5%)	1 (6%)	5 (5%)	
Kyphotic deformity > 20° at IL	50 (41%)	-	50 (48%)	
Posterior tension band injury	45 (37%)	1 (6%)	44 (42%)	
Trauma	28 (23%)	11 (65%)	17 (16%)	
SINS score ≥ 8	35 (29%)	-	35 (34%)	
**Length of instrumentation (median, range)**	4 (3–6)	1 (1–1)	5 (4–6)	***p* < 0.0001**

*p*-values in bold indicate statistical significance

**Table 3:** Complications

	**Total**	*C1/C2*	*Subaxial spine*	
** ≥ 1 surgical complication (*n*, %)**	18 (15%)	2 (12%)	16 (15%)	*p* > 0.999
Wound infection	11 (9%)	1 (6%)	10 (10%)	
Neurological worsening	3 (2.5%)	-	3 (2.8%)	
CSF fistula	2 (1.7%)	-	2 (1.9%)	
Vascular injury	3 (2.5%)	1 (6%)	2 (1.9%)	
**Second surgery due to misplaced screw (*n*, %)**	2 (1.7%)	1 (5.8%)	1 (1.0%)	*p* = 0.262
** ≥ 1 non-surgical complication (*n*, %)**	18 (15%)	1 (6%)	17 (16%)	*p* = 0.463
Pneumonia	11 (9%)	-	11 (11%)	
Urinary tract infection	10 (8%)	1 (6%)	9 (9%)	
Pulmonary embolism	3 (2.5%)	-	3 (2.9%)	
**Overall mortality (*n*, %)**	3 (2.5%)	-	3 (2.9%)	*p* > 0.999

## Discussion

In this study, we demonstrate that sagittal spinal alignment shift due to patient repositioning from supine to prone occurs frequently and across all regions of the spine, regardless of the anatomic region, underlying pathology, or the presence of morphological instability risk factors. The significance of this work is that it challenges the reliability and precision of computer-assisted spine surgery based on preoperative CT imaging alone and that caution should be used when interpreting surgical restoration of spine alignment in prone against parameters that were planned in different patient positions.

Computer-assisted spine surgery using real-time navigation, spinal robotics, or AR applications is rapidly gaining acceptance [1, 2, 8, 9, 19, 28, 30, 31]. The problem is that high costs associated with state-of-the-art intraoperative imaging limit widespread implementation and a considerable amount of time is needed for manual segmentation of AR targets acquired on intraoperative imaging. This is highly relevant, because OR time is one of the main reasons cited when spine surgeons refrain from adapting image-guidance [16]. Here, preoperative CT imaging can offset OR time and cost by allowing to plan the procedure ahead of time and extend navigation possibilities in cases where state-of-the-art intraoperative imaging is unavailable [14, 21, 25], similar to tractography for planning of cranial, neuro, and radiosurgical procedures [3, 15, 26]. Also, preoperative CT remains the foundation for machine learning-based automatic planning of pedicle screw trajectories, because preoperative CT permits consideration of pedicle shape and safety margins [23], geometry [37], and screw fixation strength based on Hounsfield units [22]. However, if preoperative data on which supporting systems are built upon is flawed then this may negatively affect the accuracy of image-guidance as well as intraoperative decision-making, for example during preoperative CT-based spinal navigation in the setting of multilevel registration or C1/C2 instability [11, 35, 40]. The present study addresses this dilemma by systematically analyzing the effect of patient repositioning between corresponding preoperative and intraoperative CT data sets on spinal sagittal alignment at the index level of a spinal pathology requiring posterior pedicle screw fixation. We deliberately focused on sagittal alignment because it seems intuitive that the sagittal plane is most likely subject to an alignment shift following patient repositioning from supine to prone. Also, we deliberately considered pathologies across the entire spine because certain levels, such as C1/C2 or the subaxial cervical spine, seem more at risk of an alignment shift, particularly in cases of instability. Our present findings of a frequent and ubiquitous spinal alignment shift mirror a recent study on cranial neurosurgery, where alternative supine and prone patient positions during preoperative magnetic resonance imaging had marked effects on lesion localization in the posterior fossa with cranial navigation [7]. That being said, the fact that odontoid fracture gap displacement or translation was merely noted in 59% of type 2 odontoid factures was unexpected, considering that alignment shift in subaxial pathologies was noted in every single case. Of course, our findings at C1/C2 need to be interpreted with caution due to the limited patient number and the fact that odontoid alignment is influenced by the reduction performed by the surgeon during patient positioning and head fixation in the 3-pin carbon fiber clamp. Still, these results indicate that not every odontoid type 2 fracture is unstable per se, which at least partially explains why navigated C1/C2 instrumentation is also feasible using preoperative CT imaging alone [11]. On the other hand, the fact that each subaxial spinal pathology exhibited at least some degree of shift clearly highlights that patient positioning needs to be considered as a likely cause for inaccuracy when using preoperative CT-based image-guidance, which argues in favor of using intraoperative 3D imaging whenever available and possible to ensure highest precision, next to the benefit of permitting direct implant control, even at the C1/C2 level [5, 17, 38].

To obtain a comprehensive view, several sagittal alignment parameters were defined and analyzed. Therefore, we also examined the general suitability of each subaxial parameter for detection of an alignment shift. Although *CA* and *PL* identified significant shift across the entire subaxial spine, the fact that the degree of shift remained uninfluenced by the region or pathology was unexpected, since we hypothesized that certain anatomic regions and pathologies like the cervical spine, traumatic fractures, or tumors with high SINS score [12] might be more prone to an alignment shift than others, such as the thoracic spine or degenerative pathologies. Possibly, this lack of difference is methodologically influenced by the fact that we measured alignment only across the region of instrumentation and not the entire spine, which could result in a higher likelihood of shift detection due to a field of view focused around the index level of pathology. On the other hand, this approach mirrors the effect that the surgeon can expect during preoperative CT-based computer-assisted surgery, which is likewise focused around the region intended for instrumentation. In any case, our findings underline that alignment shifts need to be anticipated in every case and regardless of the anatomic region or underlying pathology.

Another interesting finding was that morphological instability risk factors did not lead to a greater degree of alignment shift, although the overall high prevalence of at least one instability risk factor in our cohort (78%) could explain this result. Still, this strengthens our argument that alignment shift needs to be considered in every case and regardless of instability risk factors. The greater degree of shift in cases with an instrumentation length > 5 segments and revision cases with pre-existing instrumentation could be explained by the higher number of potentially mobile segments included in the analysis and the routine removal of implanted rods and screws intended for revision *before* the intraoperative navigation CT scan was performed, which could aggravate a sagittal alignment shift compared to shorter instrumentations and supine CT with the rods in place.

### Limitations

Although our study inherently lacks power due to its retrospective nature and single-center design, the investigated cohort is representative of patients requiring posterior spinal instrumentation for treatment of a wide spectrum of pathologies across the entire spine. Still, generalizability may be hampered due to our study design and center-specific standard operating procedures. Regarding the duration of hospitalization and complications rates, we do not believe that these were relevantly influenced by our surgical technique or misplaced screws, because our technique only included standard midline or paraspinal approaches [38] and our previous experience with iCT-based spinal navigation yielded a screw accuracy above 95% across the entire spine [18]. Also, this previously reported iCT cohort of patients was characterized by a shorter median instrumentation length (3 segments) and lower proportion of patients suffering tumor, infection, or trauma (~ 40%) than our present subgroup (~ 60%), so that a higher proportion of patients with severe comorbidities requiring more extensive surgery could explain the duration of hospitalization and complication rate that we noted in our present series. Another limitation is that we were unable to analyze spinal alignment beyond the level of instrumentation. Nevertheless, we believe that our findings also argue to use caution when interpreting appropriate surgical restoration of spine alignment achieved in prone against targeted parameters that were planned in different position [41]. Lastly, it needs to be stressed that patients in the present study did not receive an additional preoperative CT for the purpose of alignment analysis and that the available preoperative CT was either performed in an external department prior to referral or as part of an emergency algorithm. This is important because regardless of the navigation technology it adds to the total number of CT scan procedures per patient, since spinal navigation based on preoperative CT (surface matching) does not require an intraoperative scan before screw insertion and spinal navigation based on intraoperative CT (automatic co-registration as performed here) does often not require a preoperative scan [10, 34].

In conclusion, sagittal alignment shifts due to patient repositioning from supine to prone occur frequently and across all regions of the spine, which needs to be considered during computer-assisted spine surgery and intraoperative restoration of spine alignment based on parameters that were targeted in different patient positions.

## Data Availability

Raw data to support the findings of this study is available from the corresponding author upon reasonable request.
